# Treatment of anorexia nervosa in young people with autism: a literature review

**DOI:** 10.1192/bjo.2021.699

**Published:** 2021-06-18

**Authors:** Aneesa Karim

**Affiliations:** Adolescent Inpatient Unit

## Abstract

**Aims:**

The purpose of this review was to review existing literature relating to treatment of anorexia nervosa in young people with a diagnosis of autism. Hypothesis was that there would be a limited amount of literature in this age group.Previous research has suggested that there is over-representation of autistic traits in anorexia nervosa. There are implications for treatment outcomes for young people with anorexia nervosa and autism. Young people with autism may find it more difficult to engage in psychological treatments for anorexia nervosa, due to cognitive and behavioural inflexibility, or communication difficulties. Researchers are therefore looking at other options for treatment.

**Method:**

This is a narrative review. Search was conducted in January 2020. Keywords used were “anorexia nervosa” combined with “autism” combined with “treatment”. Only published, peer-reviewed, full articles in English were included. Search of OVID (for MEDLINE, PsycINFO, EMBASE and ERIC databases) gave a result of 222 articles. 9 articles met the inclusion criteria. Search of CINAHL gave a result of 12 articles; 3 articles met the inclusion criteria but had been reviewed following OVID search.

**Result:**

Themes identified for discussion were: cognitive remediation therapy; improving emotional identification; adaptations to communication; dietary, sensory and environmental considerations; recognising the role of autism; and pharmacological therapies.

**Conclusion:**

Literature suggests that treatment targeting cognitive features, common to anorexia nervosa and autism, can be effective. There has been interest in the use of cognitive remediation therapy (CRT) and cognitive remediation and emotion skills training (CREST). However, more research is required in younger patient groups. Use of medication is in experimental stages, with studies considering a role for oxytocin from age 16. Qualitative studies provide information on modifications to treatment which could be helpful. The review highlights the need for a standardised, evidence-based treatment pathway for this patient group.

